# The Metastasis-Associated Gene *MTA3,* a Component of the Mi-2/NuRD Transcriptional Repression Complex, Predicts Prognosis of Gastroesophageal Junction Adenocarcinoma

**DOI:** 10.1371/journal.pone.0062986

**Published:** 2013-05-03

**Authors:** Hongmei Dong, Hong Guo, Liangxi Xie, Geng Wang, Xueyun Zhong, Thaer Khoury, Dongfeng Tan, Hao Zhang

**Affiliations:** 1 Department of Integrative Oncology, Affiliated Cancer Hospital of Shantou University Medical College, Shantou, China; 2 Tumor Tissue Bank, Affiliated Cancer Hospital of Shantou University Medical College, Shantou, China; 3 Department of Radiation Oncology, Affiliated Cancer Hospital of Shantou University Medical College, Shantou, China; 4 Department of Thoracic Surgery, Affiliated Cancer Hospital of Shantou University Medical College, Shantou, China; 5 Cancer Research Center, Shantou University Medical College, Shantou, China; 6 Department of Pathology, Jinan University Medical College, Guangzhou, China; 7 Department of Pathology, Roswell Park Cancer Institute, Buffalo, New York, United States of America; 8 Department of Pathology, The University of Texas MD Anderson Cancer Center, Houston, Texas, United States of America; The Chinese University of Hong Kong, Hong Kong

## Abstract

Gastroesophageal junction (GEJ) adenocarcinoma carries a poor prognosis that is largely attributable to early and frequent metastasis. The acquisition of metastatic potential in cancer involves epithelial-to-mesenchymal transition (EMT). The metastasis-associated gene *MTA3,* a novel component of the Mi-2/NuRD transcriptional repression complex, was identified as master regulator of EMT through inhibition of Snail to increase E-cadherin expression in breast cancer. Here, we evaluated the expression pattern of the components of MTA3 pathway and the corresponding prognostic significance in GEJ adenocarcinoma. MTA3 expression was decreased at both protein and mRNA levels in tumor tissues compared to the non-tumorous and lowed MTA3 levels were noted in tumor cell lines with stronger metastatic potential. Immunohistochemical analysis of a cohort of 128 cases exhibited that patients with lower expression of MTA3 had poorer outcomes. Combined misexpression of MTA3, Snail and E-cadherin had stronger correlation with malignant properties. Collectively, results suggest that the MTA3-regulated EMT pathway is altered to favor EMT and, therefore, disease progression and that MTA3 expression was an independent prognostic factor in patients with GEJ adenocarcinoma.

## Introduction

Owing to an alarming increase in incidence [1], [2], gastroesophageal junction (GEJ) adenocarcinoma was recently classified as a distinct pathologic entity [3]. As it is currently defined, GEJ adenocarcinoma encompasses tumors occurring within 5 cm proximal or distal to the gastroesophageal junction [3]. GEJ adenocarcinoma is associated with a poor prognosis, with a 5-year overall survival (OS) rate of only 10–15%, largely owing to its rapid lymphatic and hematogenous metastasis [4]–[7]. Increasing evidence indicates that GEJ adenocarcinoma differs from gastric and esophageal cancers in both molecular and clinical aspects [3]. To date, mechanism of metastasis focused on GEJ adenocarcinoma is unclear and molecular markers for GEJ adenocarcinoma metastasis and tumor progression remain elusive; data from available studies are difficult to interpret because of the complexity arising from tumor heterogeneity (squamous *vs*. adenocarcinoma) and multiple primary tumor locations (esophagus, gastroesophageal junction, and others) [8]–[13].

Metastasis is a multistep event in which neoplastic cells detach from the tumor, migrate, disseminate, extravasate, and eventually proliferate at distant secondary sites. Epithelial-to-mesenchymal transition (EMT) is an initial step and an essential requirement for tumor cell spreading and carcinoma progression. The metastasis-associated gene *MTA3* is a novel component of the Mi-2/NuRD transcriptional repression complex, a major histone deacetylase [14]–[19]. All family members of metastasis tumor antigen (MTA) proteins including MTA1, MTA2, MTA3 and MTA1s have been closely linked to cancer progression and metastasis [20]. MTA3 has been described as a master regulator of EMT in human breast cancer cells [21]. Decreased levels of MTA3 result in upregulation of Snail, an event that has been identified as a trigger of EMT by causing repression of the E-cadherin cell adhesion molecule, thereby leading to a loss of cell-cell adhesion and contributing to cancer invasion and metastasis [18]. In contrast to MTA1 and MTA2, which are usually upregulated in cancer, MTA3 is downregulated [20]. Underexpression of MTA3 has been noted in tumors of the breast, endometrium, ovary, and placenta [18], [22]–[24]. Disregulation of MTA3 has been correlated with poor differentiation in endometrial cancer and poor prognosis in uterine carcinoma [22] [25].

To date, studies on MTA3 have only limited to a few types of cancers. The role of MTA3 in tumors of the gastrointestinal tract remains to be elucidated. Therefore, in this study we examined the protein expression of the EMT regulators MTA3, Snail, and E-cadherin specifically in GEJ adenocarcinoma. We then analyzed the results in conjunction with clinicopathologic parameters and survival data.

## Materials and Methods

### Patients and Tissue Samples

All specimens of primary GEJ adenocarcinoma, along with adjacent noncancerous tissue, were from patients who had undergone radical surgery without preoperative therapy at a single institution, Cancer Hospital of Shantou University Medical College, in the Chaoshan littoral, which is located in Southern China and is recognized as one of the high-incidence regions with esophageal cancer. 128 formalin-fixed paraffin-embedded surgical specimens were from patients (median age, 60 years; range, 35–81 years) who had surgery between October 2000 and October 2002. Tissues for immunoblot analysis, which were from patients who had undergone surgery between November 2009 and August 2010, were snap frozen in liquid nitrogen and stored at −80°C. All specimens were primary carcinoma that crossed the gastroesophageal junction, which defined them as GEJ adenocarcinoma according to the World Health Organization, regardless of where the bulk of the lesion was located. Resected specimens were studied in accordance with the International Union Against Cancer (UICC) pTNM classification [26].

After they were discharged, all patients returned periodically for follow-up (every 3 months for the first 3 years and every 6 months after the third year) to ensure that they did not experience disease recurrence. The median postoperative follow-up period was 29 months (range, 1–77 months). During the follow-up period, 82 patients (64%) died and 28 patients were diagnosed with distant metastasis. Written informed consents were obtained from patients in accordance with principles expressed in the Declaration of Helsinki. This study was approved by the Institutional Review Board and the Ethics Committee of Cancer Hospital of Shantou University Medical College (IRB serial number: #04–070).

### Immunoblot Analysis

MTA3 protein was assayed by immunoblot analysis in tissues lysates (80 µg of protein in RIPA lysis buffer). Protein were separated by SDS-PAGE and transferred to a PVDF membrane. The membranes were incubated in block buffer (Tris-buffered saline with 0.1% Tween and 5% nonfat dry milk) and then incubated with rabbit MTA3 antibody (1∶1000, Bethyl Laboratories, Montgomery, TX), followed by secondary antibody against rabbit IgG. Signals were visualized by ECL chemiluminescence system as described by the manufacturer (Amersham Pharmacia, NJ, USA). The blots were reprobed with anti-actin monoclonal antibody (Abcam, MA, USA) to confirm equal loading of the different samples.

### Transcript Expression Analysis

The web-based human cancer microarray database Oncomine (http://www.oncomine.org) was used to analyze the mRNA expression of MTA3 associated with esophageal and gastric adenocarcinoma in two cohorts of tumors used in previous studies [27], [28]. A comparative expression analysis of MTA3 transcripts in 39 gastric and esophageal adenocarcinoma cell lines was performed using data from the Cancer Cell Line Encyclopedia (CCLE, http://www.broadinstitute.org/ccle/home) [29].

### Immunohistochemistry and Evaluation

We performed immunohistochemical staining for E-cadherin, Snail, and MTA3 as previously described [18]. Briefly, 4-µm sections were cut from specimens that had been fixed in 10% buffered formalin and embedded in paraffin. After undergoing deparaffinization and rehydration, endogenous peroxidase blocking and antigen retrieval, specimens were incubated overnight at 4°C with anti-E-cadherin monoclonal antibody (1∶100; Santa Cruz Biotechnology, CA, USA), anti-Snail polyclonal antibody (1∶100; Santa Cruz Biotechnology, CA, USA), or anti-MTA3 polyclonal antibody (1∶200; Bethyl Laboratories, Montgomery, TX). Staining was visualized using an EnVision antibody complex method; an EnVision kit (ZSGB-BIO, Beijing, China) was employed and 3,3′-diaminobenzidine was used as the chromogen. Nuclei were counterstained with hematoxylin. Positive controls were sections from a breast cancer. Sections immunostained with rabbit or mouse IgG as the primary antibody were used as negative controls.

Immunostaining results were graded semiquantitatively by counting the percentage of positive cells and intensity. The specimens were evaluated by 2 independent observers who were unaware of the clinical information. E-cadherin immunoreactivity was assessed as follows: 1) reduced E-cadherin expression = no staining or weak-to-moderate complete membrane staining observed in >10% of tumor cells; 2) preserved E-cadherin expression = strong membrane staining in >10% of tumor cells. For evaluation of MTA3 and Snail the fraction of positively staining nuclear and cytoplasmic immunoreactivity were assigned using the following scales: 0, negative; 1+, <10% positive cells; 2+, 10–50% positive cells; 3+, >50% positive cells. Samples exhibiting 2+ and 3+ immunostaining were classified as positive expression.

### Statistical Analysis

All statistical analyses were performed using the SPSS 17.0 statistical software package (SPSS Inc., IL, USA). The statistical significance of differences in numerical data was evaluated with a Student’s t test. Correlations between MTA3 expression and clinicopathological factors were investigated using χ^2^ test. The correlation coefficient between variables was obtained using the Spearman method. Overall survival (OS) was defined as the time between surgery and time of death or the time of the last follow-up. The Kaplan-Meier method was used to assess clinical outcome after surgery. The prognostic significance of the clinical and pathological characteristics was determined using univariate Cox regression analysis. A Cox regression model for multivariate analysis was employed for factors that achieved significance in the univariate analysis. Differences were evaluated using the log-rank test. A *P*-value of less than 0.05 was considered to be significant, and all tests were 2-sided.

## Results

### Expression of MTA3 at Protein and Transcript Levels in Cancer Tissues and Cell Lines

**Figure 1:**
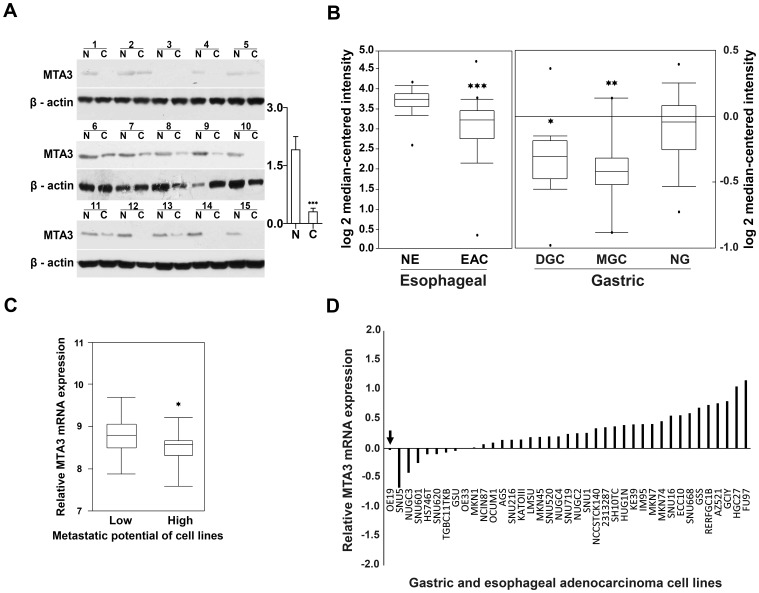
MTA3 is down-regulated in GEJ adenocarcinoma tissue and in high metastatic potential cancer cell lines. (A) MTA3 protein is down-regulated in human GEJ adenocarcinoma tissues as determined by immunoblot analysis. N, adjacent noncancerous tissues; C, cancerous tissues. (B) MTA3 transcript is significantly decreased in esophageal and gastric adenocarcinoma tissues, relative to the corresponding normal tissue. The data were from the analysis in Oncomine database. NE, normal esophagus (n = 28); EAC, esophageal adenocarcinoma (n = 75); NG, normal gastric (n = 29); GC, gastric cancer (n = 20). (C) MTA3 mRNA level is decreased in high metastatic potential cell lines of gastric adenocarcinoma and esophageal adenocarcinoma. Gene expression data for MTA3 were extracted from “CCLE Expression Entrez 2012-04-06”. (D) In all 39 tumor cell lines, the exclusively available GEJ adenocarcinoma cell line OE-19 (arrow), which has high metastatic potential, fell into the decreased-MTA3 group composed of 9 cell lines, 7 of which are more invasive. *P<0.05, **P<0.01, ***P<0.001.

Given that evidenced functions of MTA3 as a transcriptional corepressor and tumor suppressor, we determine if MTA3 is downregulated in GEJ adenocarcinoma. We examined MTA3 expression at protein level in a cohort of GEJ adenocarcinoma specimen (n = 25) by immunoblot analysis, and transcript level of MTA3 on microarray database. Immunoblot assay revealed that 72% tumor specimen had lower MTA3 protein expression than the paired adjacent noncancerous tissues (*P*<0.001; Figure 1A). In support of above findings, we analyzed MTA3 mRNA level in the human cancer microarray database Oncomine. Since there are no array data available for GEJ adenocarcinoma, we mined data from esophageal adenocarcinoma and gastric adenocarcinoma, both of which are closely relevant to GEJ adenocarcinoma. Higher MTA3 levels were noted in normal esophagus relative to esophageal adenocarcinoma (*P*<0.001; Figure 1B) [27], and in normal gastric mucosa than in mixed-type gastric cancer or diffuse-type gastric cancer (*P* = 0.008 and *P* = 0.025; Figure 1B) [28]. Moreover, mRNA expression data for MTA3 in 39 gastric and esophageal adenocarcinoma cell lines were extracted from CCLE (http://www.broadinstitute.org/ccle/home) [29]. MTA3 mRNA expression appeared more common in low metastatic cell lines than in cell lines with high metastatic potential (*P* = 0.035; Figure 1C). Interestingly, OE-19, the exclusive GEJ adenocarcinoma cell line which has high metastatic potential, had lower levels of MTA3 mRNA expression compared to most other gastric and esophageal adenocarcinoma cell lines (Figure 1D arrow). Accordingly, these results consistently indicate that MTA3 is downregulated in tumorous tissues, and underexpression of MTA3 is related with stronger metastasis tendency in tumor cells, supporting the hypothesis that MTA3 may act as a tumor suppressor and metastasis inhibitor in GEJ adenocarcinoma.

### Expression of MTA3, Snail, and E-cadherin in GEJ Adenocarcinoma

**Figure 2:**
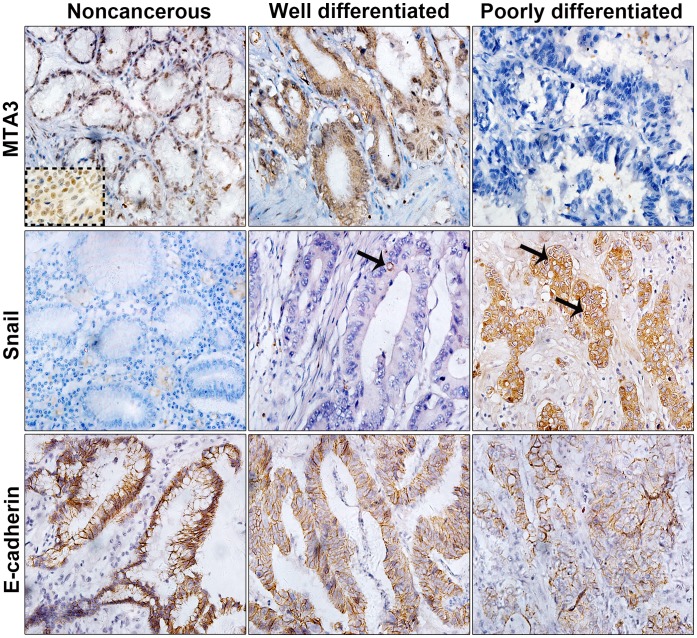
Immunohistochemical staining for MTA3, Snail, and E-cadherin in GEJ adenocarcinoma and adjacent noncancerous tissue. Representative samples of noncancerous tissue, well differentiated tumor and poorly differentiated tumor are shown. Strong to negative staining for MTA3 (top). Negative to strong staining for Snail (middle). Strong to weak staining for E-cadherin (bottom). The arrow indicates perinuclear staining of Snail. The dotted insert showed strongly positive staining for MTA3 in breast cancer cells as positive control (original magnification 400×).

MTA3 has been recognized as a master inhibitor of EMT in cancer, which inhibits Snail to increase E-cadherin expression. To obtain the functional insight into MTA3 in EMT and tumor progression of GEJ adenocarcinoma, we characterized the expression profile of MTA3-pathway components in GEJ adenocarcinoma. Protein expression of MTA3, Snail and E-cadherin was assayed in a cohort of 128 primary GEJ adenocarcinoma. In total examined specimens, positive immunostaining for MTA3 was observed in both the nucleus and the cytoplasm of neoplastic cells in 63 (49.2%), whereas strong MTA3 immunoreactivity was predominantly found in the nuclei of epithelial cells in noncancerous tissues (Figure 2; top). Human breast cancer samples served as a positive control for MTA3 immunoreactivity (dotted insert in Figure 2). Tumor cells in 87 (68%) of the specimens exhibited strong cytoplasmic staining for Snail; in contrast, there was an absence of staining for Snail in all the noncancerous epithelial cells (Figure 2; middle). Perinuclear staining was frequently observed in Snail-positive cells (arrows in Figure 2). In contrast to the noncancerous epithelial cells, which exhibited strong E-cadherin immunostaining, decreased membrane staining for E-cadherin was observed in tumor cells in 73 (57%) of the specimens (Figure 2; bottom). Taken together, these observations clearly suggest that MTA3-regulated EMT pathway is altered in GEJ adenocarcinoma.

### MTA3 Expression and Clinicopathologic Variables

We next assessed the correlation of MTA3 expression with the clinicopathological features in GEJ adenocarcinoma. MTA3 expression was associated with a histologic differentiation of well or moderately differentiated adenocarcinoma (*vs.* poorly differentiated; *P* = 0.004), stage I or II disease (*vs.* stage III disease; *P*<0.001), a lack of lymph node metastasis (*P* = 0.001), a lack of distant metastasis (*P* = 0.013), decreased Snail expression (*P*<0.001), and increased E-cadherin expression (*P*<0.001; Table 1). The expression level of MTA3 was inversely correlated with that of Snail (*r* = -0.323; *P*<0.001) and was positively correlated with that of E-cadherin (*r* = 0.629; *P*<0.001) (data not shown).

**Table 1:** Relationship between MTA3 expression and clinicopathologic variables in tissue samples of GEJ adenocarcinoma (n = 128).

Variables	No. of samples	MTA3 expression		*P* value
		Negative, no. (%)	Positive, no. (%)	
All samples	128	65 (50.8)	63 (49.2)	
Sex				
Male	106	51 (48.1)	55 (51.9)	0.185
Female	22	14 (63.6)	8 (36.4)	
Age (years)				
≤60	65	28 (43.1)	37 (56.9)	0.077
>60	63	37 (58.7)	26 (41.3)	
Tumor size				
<5 cm	64	29 (45.3)	35 (54.7)	0.216
≥5 cm	64	36 (56.3)	28 (43.7)	
Histologic differentiation				
Well/Moderate	51	18 (35.3)	33 (64.7)	0.004
Poor	77	47 (61.0)	30 (39.0)	
General type				
Infiltrating-ulcerative	62	35 (56.5)	27 (43.5)	0.578
Ulcerative	50	22 (44.0)	28 (56.0)	
Infiltrating	9	5 (55.6)	4 (44.4)	
Others	7	3 (42.9)	4 (57.1)	
Tumor depth				
T_1_/T_2_	9	3 (33.3)	6 (66.7)	0.278
T_3_/T_4_	119	62 (52.1)	57 (47.9)	
Lymph node metastasis				
N0	43	13 (30.2)	30 (69.8)	0.001
N1	85	52 (61.2)	33 (38.8)	
Distant metastasis				
M0	100	45 (45.0)	55 (55.0)	0.013
M1	28	20 (71.4)	8 (28.6)	
Stage				
I/II	43	11 (25.6)	32 (74.4)	<0.001
III	85	54 (63.5)	31 (36.5)	
E-cadherin expression				
Negative	73	57 (78.1)	16 (21.9)	<0.001
Positive	55	8 (14.5)	47 (85.5)	
Snail expression				
Negative	41	11 (26.8)	30 (73.2)	<0.001
Positive	87	54 (62.1)	33 (37.9)	

An analysis of the combined expression patterns of the 3 proteins revealed that the presence of MTA3-negative/Snail-positive/E-cadherin-negative tumors (*vs.* other expression patterns) was associated with lymph node metastasis (*P* = 0.018), stage III disease (*vs.* stage I or II; *P* = 0.002), and a histologic differentiation (*P = *0.008; Table 2). In summary, our results demonstrate that misexpression of MTA3-pathway components is closely associated with parameters involving invasion/metastasis, and tumor advancement. Thus, these data provide favorable evidence that the altered MTA3 pathway contributes to the EMT and tumor metastasis, and thereby the tumorigenic process of GEJ adenocarcinoma.

**Table 2:** Relationship between combined expression patterns of MTA3, Snail, and E-cadherin (MTA3−/Snail+/E-cadherin- *vs.* other expression patterns) and clinicopathologic variables in tissue samples of GEJ adenocarcinoma (n = 128).

Variables	MTA3−/Snail+/E-cadherin-, no. (%)	Other patterns, no. (%)	*P* value
All samples	48 (37.5%)	80 (62.5%)	
Sex			
Male	42 (39.6)	64 (60.4)	0.276
Female	6 (27.3)	16 (72.7)	
Age (years)			
≤60	22 (33.8)	43 (66.2)	0.386
>60	26 (41.3)	37 (58.7)	
Tumor size			
<5 cm	21 (32.8)	43 (67.2)	0.273
≥5 cm	27 (42.2)	37 (57.8)	
Histologic differentiation			
Well/Moderate	12 (23.5)	39 (76.5)	0.008
Poor	36 (46.8)	41 (53.2)	
Tumor depth			
T_1_/T_2_	2 (22.2)	7 (77.8)	0.326
T_3_/T_4_	46 (38.7)	73 (61.3)	
Lymph node metastasis			
N0	10 (23.3)	33 (76.7)	0.018
N1	38 (44.7)	47 (55.3)	
Distant metastasis			
M0	34 (34.0)	66 (66.0)	0.122
M1	14 (50.0)	14 (50.0)	
Stage			
I/II	8 (18.6)	35 (81.4)	0.002
III	40 (47.1)	45 (52.9)	

### Relationship between OS and the Expression of MTA3, Snail, and E-cadherin

**Figure 3:**
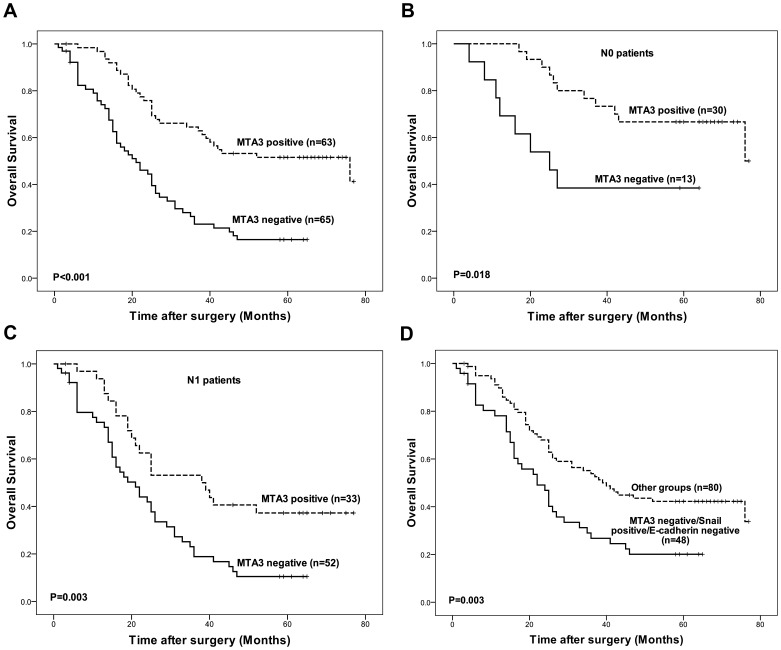
Survival curves of patients according to expression statues of MTA3. (A) The OS was significantly better among the patients with MTA3-positive tumors than among the patients with MTA3-negative tumors (*P*<0.001). (B) Among the patients with no lymph node metastasis (N0), the OS was significantly better in the patients with MTA3-positive tumors than in the patients with MTA3-negative tumors (*P* = 0.018). (C) Among the patients with lymph node metastasis (N1), the OS was significantly better in the patients with MTA3-positive tumors than in the patients with MTA3-negative tumors (*P* = 0.003). (D) The OS among the patients with MTA3-negative/Snail-positive/E-cadherin-negative tumors was significantly worse than among the patients whose had tumors exhibited other expression patterns (*P* = 0.003).

To evaluate the prognostic impacts of MTA3-pathway components on the outcomes of patients with GEJ adenocarcinoma, we performed Kaplan-Meier survival analyses. The OS rate of patients with tumors that expressed MTA3 was significantly higher than that of patients with tumors that did not express MTA3 (*P*<0.001; Figure 3A). An analysis of the relationship between MTA3 expression and OS was performed separately in patients with lymph node metastasis (N1) and in patients without lymph node metastasis (N0). In both groups, the OS rate was higher in patients with tumors that expressed MTA3 than in patients with tumors that did not express MTA3 (*P = *0.018 for N0 and *P = *0.003 for N1; Figure 3B and 3C). In subgroup of patients with MTA3-negative/Snail-positive/E-cadherin-negative tumors, the OS rate was lower than in patients who had tumors with other expression patterns (*P* = 0.003; Figure 3D). Thus, the analysis indicates that expression of MTA3-pathway components is strongly associated with the survival of GEJ adenocarcinoma patients.

### Univariate and Multivariate Analysis of OS

To test possibility that MTA3 may be served as a prognostic predictor for GEJ adenocarcinoma patients, we applied univariate and multivariate Cox-regression models for further analysis. In the univariate analysis, male sex (*P* = 0.026), stage III disease (*vs.* stage I or II; *P* = 0.001), lymph node metastasis (*P* = 0.001), distant metastasis (*P* = 0.001), a lack of E-cadherin expression (*P* = 0.001), Snail expression (*P* = 0.006), and loss of MTA3 expression (*P*<0.001) were associated with decreased OS (Table 3). An increased risk of death was also observed among subgroup of patients with MTA3-negative/Snail-positive/E-cadherin-negative tumors compared with patients who had tumors exhibited other expression patterns (*P* = 0.004; Table 3). However, the multivariate analysis indicated that lymph node metastasis (HR 2.185; 95% CI 1.276 to 3.740; *P* = 0.004), male sex (HR 1.782; 95% CI 1.061 to 2.993; *P* = 0.029), and MTA3 expression (HR 0.442; 95% CI 0.275 to 0.708; *P* = 0.001) were independent prognostic factors (Table 3).

**Table 3:** Univariate and multivariate Cox proportional hazards models showing variables that affect overall survival in patients with GEJ adenocarcinoma.

Characteristic	Univariate analysis		Multivariate analysis	
	HR (95% CI)	*P* value	HR (95% CI)	*P* value
Sex				
Female *vs*. Male	1.798 (1.073–3.012)	0.026	1.782 (1.061–2.993)	0.029
Stage				
I/II *vs*. III	2.353 (1.417–3.907)	0.001	1.246 (0.677–2.291)	0.480
Lymph node metastasis				
N0 *vs*. N1	2.676 (1.593–4.495)	0.001	2.185 (1.276–3.740)	0.004
Distant metastasis				
M0 *vs*. M1	2.249 (1.401–3.608)	0.001	1.466 (0.879–2.444)	0.143
E-cadherin expression				
positive *vs*. Negative	0.471 (0.297–0.747)	0.001	0.775 (0.403–1.492)	0.446
Snail expression				
Negative *vs*. positive	2.798 (1.346–5.815)	0.006	1.454 (0.747–2.829)	0.271
MTA3 expression				
positive *vs*. Negative	0.354 (0.224–0.558)	<0.001	0.442 (0.275–0.708)	0.001
Combination of MTA3/Snail/E-cadherin				
Others *vs*. MTA3−/Snail+/E-cadherin-	0.521 (0.335–0.810)	0.004	1.483 (0.611–3.596)	0.384

HR hazard ratio, CI confidence interval.

## Discussion

In this study, we discovered that expression of MTA3-pathway components was altered in tumor tissues, and the altered expression was strongly correlated with metastasis and tumor advancement. Our results indicate that the MTA3-regulated EMT pathway is altered to favor EMT in GEJ adenocarcinoma and that MTA3 expression is an independent prognostic factor in patients with GEJ adenocarcinoma.

The immunostaining patterns of EMT-related proteins Snail and E-cadherin observed in GEJ adenocarcinoma are similar to the previously reported staining patterns of Snail and E-cadherin in gastric and esophageal cancers [8], [9], [11], [12], [30], [31]. The frequent lack of MTA3 expression in GEJ adenocarcinoma that we observed is consistent with its essential role in EMT as reported in breast, endometrial, and ovarian cancers [15], [18], [22], [24], [25].

Reduced MTA3 expression has been reported to be associated with poor differentiation in endometrioid adenocarcinomas [22] but not with any clinicopathologic indicators of ovarian cancer [24]. However, we found that, in GEJ adenocarcinoma, the loss of MTA3 was associated with several clinicopathologic indicators, including poor tumor differentiation, advanced disease stage, lymph node and distant metastasis, increased Snail expression, and decreased E-cadherin expression. Of these clinicopathologic factors, metastasis appears to be an important prognostic factor in GEJ adenocarcinoma [3], [32]–[34]. In support of the role of MTA3 in metastasis, our analysis of MTA3 transcript in gastric and esophageal adenocarcinoma cell lines on CCLE identified a strongly inverse correlated tendency between MTA3 expression and the metastatic potential of the tumor cells.

We found that MTA3 expression was a strong independent prognostic factor for favorable OS. Similarly, another recent study demonstrated that MTA3 was an independent prognostic indicator in uterine carcinoma [25]. Although Snail and E-cadherin are critical for EMT, neither the absence of Snail expression nor the presence of E-cadherin expression alone was associated with improved OS in the multivariate analysis. This result indicates that MTA3, as a master coregulator, may influence multiple critical target molecules, such as Snail and E-cadherin, that control EMT and other processes associated with disease progression. Interestingly, there was a significantly increased risk of death among subgroup of patients with tumors that were MTA3-negative/Snail-positive/E-cadherin-negative compared with patients who had tumors with other expression patterns, reflecting the joint contribution of multiple EMT components in the disease progression.

Because patients usually present with locally advanced disease, biomarkers other than lymph node status may be needed to predict patient outcome. In this study, a lack of MTA3 expression was also associated with poor OS in patients without lymph node metastasis. This result suggests that MTA3 may be of value in predicting the outcomes of patients with early-stage disease.

MTA3 was initially identified in breast cancer as an estrogen receptor (ER)-regulated inhibitor involved in the ER-dependent downregulation of the Snail-mediated inhibition of E-cadherin [14]. To date, studies on MTA3 expression have been limited to a few hormone-responsive malignancies, such as tumors of the breast, ovary, endometrium, and placenta. However, because MTA3 is a newly described member of the MTA family, only a few of its targets have been identified. Our previous study in MTA3 transgenic mice suggested that MTA3 expression is not necessarily related to ER status [17], raising the possibility that MTA3 may act as a regulator within a hormone-independent context [25]. The results of the present study suggest that the MTA3 inhibition of Snail and the subsequent regulation of E-cadherin expression also occur in hormone-independent cancers.

There are some limitations generalizing the results of this study. Extension of our study to larger sample size may be needed. In addition, our findings, which were derived from examination of patient specimens, remain to be validated under well-controlled conditions in cell cultures and animal models.

Taken together, the results suggest that MTA3 and the EMT pathway regulators Snail and E-cadherin have a substantial role in the metastasis and progression of GEJ adenocarcinoma. These regulators may act individually or cooperatively. Our results also suggest that MTA3 expression is independent prognostic factors in GEJ adenocarcinoma. Further evaluation of the MTA3-modulated EMT pathway may increase our ability to assess prognosis in patients with GEJ adenocarcinoma.
